# NEDD4 and NEDD4L: Ubiquitin Ligases Closely Related to Digestive Diseases

**DOI:** 10.3390/biom14050577

**Published:** 2024-05-13

**Authors:** Jiafan Xu, Wang Jiang, Tian Hu, Yan Long, Yueming Shen

**Affiliations:** Department of Digestive Diseases, The Affiliated Changsha Central Hospital, Hengyang Medical School, University of South China, 161 Shaoshan Road, Changsha 410000, China; xjf2012196436@163.com (J.X.); jiangwang0205@163.com (W.J.); 15874932410@163.com (T.H.); jenny199301@163.com (Y.L.)

**Keywords:** nedd4, nedd4l, ubiquitin ligases, ubiquitin–proteasome system, cancer, digestive system diseases

## Abstract

Protein ubiquitination is an enzymatic cascade reaction and serves as an important protein post-translational modification (PTM) that is involved in the vast majority of cellular life activities. The key enzyme in the ubiquitination process is E3 ubiquitin ligase (E3), which catalyzes the binding of ubiquitin (Ub) to the protein substrate and influences substrate specificity. In recent years, the relationship between the subfamily of neuron-expressed developmental downregulation 4 (NEDD4), which belongs to the E3 ligase system, and digestive diseases has drawn widespread attention. Numerous studies have shown that NEDD4 and NEDD4L of the NEDD4 family can regulate the digestive function, as well as a series of related physiological and pathological processes, by controlling the subsequent degradation of proteins such as PTEN, c-Myc, and P21, along with substrate ubiquitination. In this article, we reviewed the appropriate functions of NEDD4 and NEDD4L in digestive diseases including cell proliferation, invasion, metastasis, chemotherapeutic drug resistance, and multiple signaling pathways, based on the currently available research evidence for the purpose of providing new ideas for the prevention and treatment of digestive diseases.

## 1. The Ubiquitin–Proteasome System (UPS)

**Figure 1:**
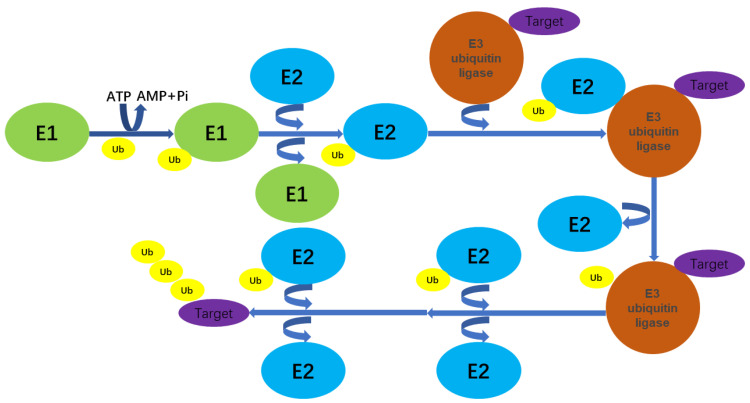
Ubiquitination process: First, E1 activates ubiquitin by attaching to the Cys residue in the tail of the ubiquitin molecule in the ATP energy supply. Then, E1 transfers the activated ubiquitin molecule to E2, which subsequently recognizes the target protein and modifies it through ubiquitination, along with a number of special E3 enzymes.

The ubiquitin–proteasome system (UPS) is a major protein hydrolysis system in eukaryotes [1], regulating a range of life processes such as cell cycle regulation, apoptosis, inflammation, transcription, and signaling, and has been closely associated with the development of multiple diseases [2,3]. A better understanding of the UPS process can help to clarify the pathogenesis of these diseases and therefore develop more efficient drugs based on the mechanisms identified [4,5]. Ubiquitin is a small protein consisting of 76 amino acids, with a molecular weight of approximately 8.451 kDa. It is present in most eukaryotic organisms and appears to be highly conserved in terms of evolution [6,7]. Ubiquitin contains seven lysine (Lys) sites (K6, K11, K27, K29, K 33, K48, and K63), a methionine (Met) site at the N-terminal (M1), and a glycine (Gly) site at the C-terminal (G76) [7,8,9]. Ubiquitin makes various connections between ubiquitin, mainly through lysine residues and methionine residues, and the resulting ubiquitin chain modifies the protein substrate, which encodes information that can be deciphered by effector proteins with linkage-specific ubiquitin-binding structural domains (UBDs) to trigger a specific biological outcome [7,8,10]. Ubiquitination, a key mechanism of proteasome-mediated protein degradation, is the process by which ubiquitin, in the presence of a series of enzymes, sorts and labels intracellular proteins to screen target protein molecules before modifying the target protein specifically [2,9]. Ubiquitination is involved in the regulation of almost all life activities such as the cell cycle [11], proliferation, apoptosis, differentiation, metastasis, gene expression, transcriptional regulation, signaling, damage repair, and inflammation immunity [8,12]. The side chain of the surface lysine of the substrate protein is the only amino acid containing an ε-amino group with a range of primary amine structural features and reaction properties involved in a variety of post-translational modifications in vivo [11,13,14]. When the ε-amino group of a lysine residue in a target protein binds a single ubiquitin molecule, this is referred to as mono-ubiquitination and is a one-to-one relationship. When multiple Lys residues of a target protein are simultaneously tagged by a single ubiquitin molecule, this is poly-ubiquitination. When a single Lys residue of a target protein is tagged by multiple ubiquitin molecules, this is poly-ubiquitination and is a many-to-one relationship [8,15,16]. The method by which the ubiquitination chains on the substrate proteins are attached and the sites where ubiquitination occurs are the key factors in protein signal transduction [7,8]. For example, K48 ubiquitin chain linkage is the most abundant linkage between ubiquitin molecules in cells and was initially known as a typical signal for the degradation of target proteins [7,17]. The K63 ubiquitin chain has been found to regulate the activation of the transcription factor NF-κB [18,19], DNA repair [20,21], the innate immune response [22], and to degrade target substrates through autophagy [23,24,25]. The K11 ubiquitin chain has been revealed to participate in receptor transport, modification of cell cycle regulatory proteins, DNA repair, and signal transduction [26,27]. The K6 ubiquitin chains may be associated with DNA damage, mitochondrial autophagy, etc. [28,29]. The K27-linked ubiquitin chains may serve as scaffolds for protein recruitment in the response to DNA damage [26,30]. The K29 ubiquitin chains may regulate lysosomal degradation [31,32] and the K33 chains have been implicated in a variety of biological processes [33,34]. The ubiquitination of proteins is a multistep process that involves three enzymes, namely ubiquitin-activating enzyme (E1), ubiquitin-conjugating enzyme (E2), and ubiquitin ligase (E3). First, E1 activates ubiquitin by attaching to the Cys residue in the tail of the ubiquitin molecule in the ATP energy supply [35]. Then, E1 transfers the activated ubiquitin molecule to E2, which subsequently recognizes the target protein and modifies it through ubiquitination, along with a number of special E3 enzymes [36,37,38] (Figure 1).

E3 ubiquitination ligases are the determinants of target-protein-specific recognition [39]. To date, 600 E3 ligases have been identified in the human genome and are divided into three main families, namely the RING family, the HECT family, and the inter-loop RBR family [40,41]. Among the three families, HECT plays the most important role [42,43]. It contains a 350-amino-acid C-terminus with a cysteine site, whereas the N-terminus can bind to E2 ligases and partially determines the substrate specificity, Therefore, the HECT family can be further divided into three subfamilies: the neuronal precursor cell-expressed developmentally downregulated 4 (NEDD4) subfamily, which contains a tryptophan-tryptophan acid (WW) motif; the HECT and RCC-like structural domain (HERC) subfamily, which has one or more chromosome condensation 1 (RCC 1)-like structural domains; and other HECTs, which contain a variety of domains [44,45,46].

**Figure 2:**
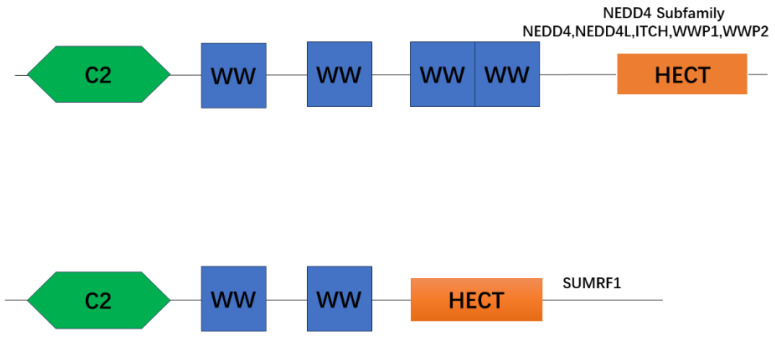
The NEDD4 family has three functional structural domain parts: the N-terminal C2 structural domain for lipid membrane binding (green), the central 2-4 WW structural domains involved in interactions with substrates (blue), and the C-terminal HECT catalytic structural domain associated with E3 ubiquitin ligase activity (orange).

More specifically, NEDD4 is the largest HECT E3 subfamily [47]. In lactating animals, this subfamily contains nine members, namely NEDD4-1/NEDD4, NEDD4-2/NEDD4L, Smad-specific E3 ubiquitin protein ligases 1 (SMURF 1) and 2 (SMURF 2), WW domain-containing E3 ubiquitin protein ligases 1 (WWP 1) and 2 (WWP 2), ITCH, NEDL1/HECW1, and NEDL2/HECW 2 [46,48,49] (Figure 2).

NEDD4 and NEDD4L are the earliest discovered members of the NEDD4 subfamily and are the most frequently studied proteins for the time being [50]. NEDD4 and NEDD4L are 900-amino-acid proteins containing several folded structural domains: (1) the N-terminal C2 structural domain, which is a 116-amino-acid calcium-dependent lipid-binding region that plays a role in protein–protein interactions by binding to Ca 2+ to induce the translocation of NEDD4-bound phosphatidylcholine to the plasma membrane [36,51]; (2) the four WW structural domains which recognize PPR motifs and allow for cellular localization and substrate recognition, playing a role in regulating protein–protein interactions [52,53]; (3) the conserved cysteine residue in the structural domain of the C-terminal HECT ubiquitin ligase, which generates an intermediate thioester bond with the active ubiquitin received from E2 and catalyzes the ligase activity of the transfer of Ub from E2 to the substrate [51,54].

The NEDD4 gene, located on human chromosome 15q21.3, is commonly expressed in a variety of human organs and tissues (e.g., skin, liver, thyroid, kidney, and vascular endothelial cells) [40,55,56] and has been associated with the development of a variety of diseases, including cancer, cardiovascular diseases, metabolic diseases, neurological diseases, renal diseases, digestive diseases, etc. [48,55,57,58]. For example, NEDD4 is overexpressed in a number of cancers (e.g., breast, bladder, and colon cancer) [59,60,61,62], where it can perform its oncogenic or oncostatic function by inducing the ubiquitination of PTEN, Myc, Ras, and other substrates [63,64,65]. The NEDD4L gene, located on human chromosome 18 q21, contains the WW and HECT structural domains [66]. It regulates a variety of membrane proteins and acts on multiple ion channels, including chloride, potassium, and sodium ions, and it exerts a significant effect on the development of the central nervous system and the regulation of hypertension [67,68,69,70,71]. Moreover, NEDD4L is also involved in Acquired Immune Deficiency Syndrome (AIDS) [23]. It directly catalyzes the K29-linked ubiquitination of tumor necrosis factor receptor-acting factor 3 (TRAF3), which regulates TRAF3 activity to promote innate immunity with antiviral effects [23] and plays a role in Wnt signaling, cellular autophagy, and pulmonary fibrosis [72,73,74].

In recent years, the relationship between NEDD4/NEDD4L and digestive diseases has become a hotspot research topic. The current results show that NEDD4 and NEDD4L can regulate digestive system functions and a series of related physiological and pathological processes via multiple pathways. In this paper, we reviewed the roles of NEDD4 and NEDD4L in various digestive diseases for the purpose of providing new strategies for the prevention and treatment of digestive diseases.

## 2. NEDD4/NEDD4L in Digestive System Diseases

### 2.1. NEDD4/NEDD4L and Gastrointestinal Disorders

#### 2.1.1. NEDD4/NEDD4L and Esophageal Cancer

Esophageal cancer is the seventh most common cancer worldwide and the sixth most common cause of cancer-related deaths [75,76]. Esophageal squamous cell carcinoma (ESCC) is a common subtype of esophageal cancer with a high incidence [77,78]. Currently, the most important treatment for ESCC is early surgery, but the treatment outcomes are not satisfactory [79,80]. In a recent study, Wei Cheng et al. found that NEDD4L was significantly reduced in ESCC specimens and was associated with poor clinical prognoses. The results suggest that NEDD4L is able to inhibit cell viability, cell cycle progression, and glutamine metabolism in ESCC by inducing the ubiquitination degradation of c-Myc and reducing the expression of glutaminase (GLS1) and solute carrier family 1 member 5 (SLC1A5). This finding provides new ideas for the treatment of ESCC [81]. Because esophageal cancer has a poor prognosis, we need to actively study the pathogenesis of esophageal cancer and develop new drugs for the treatment of ESCC to improve patient outcomes (Table 1).

#### 2.1.2. NEDD4/NEDD4L and Gastric Cancer

**Figure 3:**
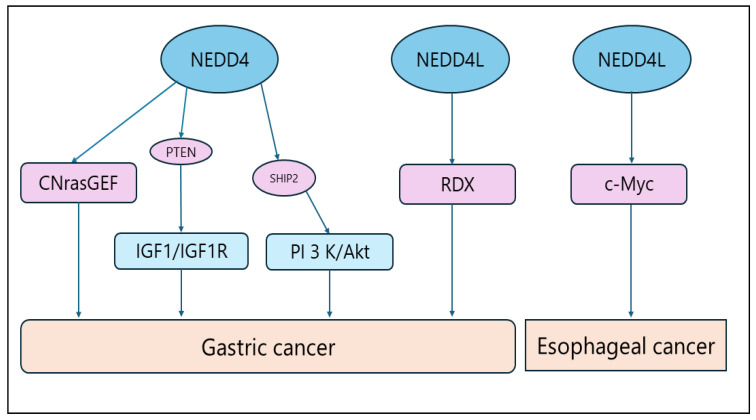
NDDD4/NDDD4L signaling pathway in esophageal and gastric cancer.

Gastric cancer (GC) is the fifth most common malignant tumor in the world and the third most common cause of cancer-related deaths [114,115]. Its pathogenesis is extremely complex and involves a variety of causes, such as geographic environment and dietary factors, heredity and mutation, helicobacter pylori (HP) infection, and precancerous lesions, etc. [116]. It is noteworthy that most patients with GC do not have obvious symptoms in the early stages of the disease [117,118]. An earlier study found that NEDD4 was overexpressed in GC [119] and was associated with the phosphatase and tensin homolog (PTEN) [89]. Even though many scholars argue that NEDD4 is a potential dual regulator of PTEN, the relationship between NEDD4 and PTEN remains divergent. For example, Zhen Yang et al., reported that NEDD4 and PTEN were differentially expressed in all stages of gastric carcinogenesis. Specifically, the difference was that PTEN expression in primary tumors was significantly lower than in neighboring non-tumor tissues, while there was no difference in the expression of NEDD4, indicating that the expression levels of NEDD4 and PTEN are not correlated with each other during gastric carcinogenesis. Thus, the effect of NEDD4 on the regulation of PTEN function during gastric carcinogenesis remains to be clarified in different stages of GC development. Accordingly, the regulatory role of NEDD4 on PTEN function in GC development should also be further investigated with larger samples [120,121]. In another study, Aiqin Sun and colleagues examined the expression of NEDD4 in 214 GC tissue specimens and found that NEDD4 was overexpressed in 83% of the total sample. It was also shown that NEDD4 gene knockdown significantly reduced the migratory and invasive ability of GC cells, suggesting that NEDD 4 may be a biomarker for determining the prognosis of gastric cardia adenocarcinoma (GCA) among GC types, and that NEDD4 may be a new target for antimetastatic GC therapy [122]. DONG LI et al. found that decitabine (DAC) promoted the cell invasion and metastasis of GC cell line MGC 803 by mediating the ubiquitination degradation of the cyclic nucleotide-Ras guanine nucleotide exchange factors (CNrasGEFs) through upregulation of the expression of NEDD4 but had no effect on the invasive properties of another cell line, SGC 7901. This finding affirms the prognostic value of NEDD4 in GC and opens up a new way of thinking about the clinical application of DAC [82]. In clinical practice, 5-Fluorouracil (5-Fu) is generally used as the first-line chemotherapy drug for patients with advanced GC [86,123]. It can prolong the patient’s survival, but due to drug resistance, patients at later stages often continue to experience recurrent disease. Yifan Lu et al. found that calponin 1 (CNN 1) was highly expressed in cancer-associated fibroblasts (CAFs) in GC tissues and predicted that GC patients taking 5-Fu treatment had a poor prognosis. The authors reported that CNN 1 interacted with PDZ and LIM Domain 7 (PDLIM 7) and prevented its degradation by NEDD4, which would activate the ROCK1/MLC pathway to induce the contraction of CAFs and increase matrix stiffness, thereby leading to 5-Fu resistance in GC cells following activation of the Yes protein (YAP). This suggests that targeting CAFs may overcome GC resistance and improve the therapeutic efficacy of radiotherapy for GC [86,87]. Ke Wang et al. found that NEDD4 overexpression promoted tumor proliferation in insulin-like growth factor 1 receptor (IGF1R)-dependent GC cells, and therefore hypothesized that NEDD4 specifically promotes the growth of GC cells that are dependent on the IGF1/IGF1R signaling pathway by antagonizing the protein phosphatase activity of IRS 1 by antagonizing PTEN. Thus, NEDD4 may be one of the therapeutic targets for the treatment of GC driven by the IGF 1 signaling pathway [83,84]. Recently, Liang Xu et al. reported that the expression of inhibin 2 (PHB 2) in GC tissues was significantly higher than in normal tissues adjacent to the cancer and was associated with poor clinical prognosis. The results suggest that PHB 2 can promote the ubiquitination degradation of SHIP 2 by increasing the interaction between NEDD4 and SHIP2, thereby activating the PI3K/Akt signaling pathway to promote GC cell proliferation [85]. In addition, Xingwang Jiang and colleagues detected the mRNA expression of NEDD4L and HIF-1α in both fresh GC and normal control tissues and found that the expression of NEDD4L was significantly downregulated while the expression of HIF-1α was upregulated in GC. For this reason, it was speculated that NEDD4L and HIF-1α are independent prognostic factors for GC, and the combined expression of NEDD4L and HIF-1α is an important indicator for judging the prognosis of GC patients. Although the exact molecular mechanism has not been fully clarified yet, it may facilitate the development of new anticancer strategies [124]. Xiaocheng Tang et al. revealed that intercellular adhesion molecule 2 (ICAM 2) was significantly downregulated in GC cells and tissues and could be used to evaluate prognoses, and that ICAM 2 promoted the ubiquitination degradation of radixin (RDX) in a NEDD4L-dependent manner to inhibit GC cell proliferation and migration [88]. Overall, the above evidence suggests that NEDD4 and NEDD4L are involved in the development of GC via a variety of signaling pathways, but some of the molecular mechanisms are still uncertain and require further study (Figure 3) (Table 1). 

#### 2.1.3. NEDD4/NEDD4L and Colorectal Cancer

**Figure 4:**
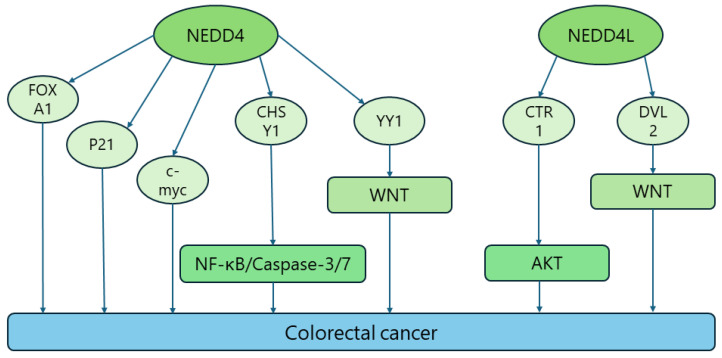
NDDD4/NDDD4L signaling pathway in colorectal cancer.

Colorectal cancer (CRC) is a common intestinal tumor and the third leading cause of cancer-related deaths worldwide [125]. Its pathogenesis is complex and has not yet been fully elucidated, and the majority of patients present with symptoms at a late stage. Therefore, we need to improve the diagnosis, early detection, and early treatment of CRC [126,127]. Current NEDD4 family members in the ubiquitinated proteasome system, especially NEDD4 and NEDD4L, are currently being studied by many scholars for the purpose of elucidating their roles in CRC. Sung Soo Kim et al. found that NEDD4 was expressed in 80% of CRC cases and played an oncogenic role by inducing ubiquitinated degradation of PTEN [89]. Peter W and colleagues investigated whether NEDD4 expression was associated with the PTEN mutation status in 114 CRC cases, and revealed that NEDD4 knockdown would not affect PTEN protein levels or the PI3K/AKT signaling pathway, indicating that the regulation of PTEN by NEDD4 is not stable and NEDD4 can independently drive CRC development without regulating PTEN levels [59]. Meng Yue et al. found that NEDD4 could promote the development of CC by targeting the ubiquitination degradation of forkhead box A1 (FOXA1). FOXA1 would then bind to the promoter region of microRNA-340-5p, which then binds to the mRNA of activating transcription factor 1 (ATF1) to induce the downregulation of ATF1, thus blocking some of the oncogenic functions of NEDD4 in CC cells. This finding has provided a better understanding of the pathogenesis of CC and a new idea for the treatment of CC [90]. Similarly, Sen Zhang and colleagues reported that the expression of N-myc downstream-regulated gene 1 (NDRG1) was downregulated in CRC tissues, and that NDRG1 could suppress the proliferation of colorectal tumors by inhibiting the NEDD4-mediated ubiquitination degradation of P21, suggesting that NDRG1 may be a potential anti-oncogene in CRC development. This unveils a new target for the treatment of CRC [94] (p. 21). Recently, Ying-Nan Wang et al. cultured CRC cells with cholesterol-lowering agents and lipoprotein-free medium, and found that SR10 [84], a RAR-related orphan receptor α/γ (RORα/γ) agonist, could activate the transcription of NEDD4 by binding to the promoter of the NEDD4 gene, thus promoting the ubiquitylation and degradation of c-myc and inhibiting the proliferation and metastasis of CRC cells. Moreover, atorvastatin combined with ROR α/γ agonists was found to have a synergistic effect in inhibiting the proliferation and migration of CRC cells, which provides a new strategy for CRC therapy by inhibiting the cholesterol-ROR α/γ–c-myc axis [91]. Shaojun Yu et al. reported that the expression level of microchromosome maintenance 8 homologous recombination repair factor (MCM8) was upregulated in CRC tissues and was significantly correlated with the tumor grade and prognosis, and that MCM8 knockdown could inhibit the growth of CRC cells. This suggests that MCM8 can act as a tumor promoter in CRC and may be regarded as a promising therapeutic target for CRC [93]. MCM8 plays a role in regulating CHSY1 protein stability by affecting the NEDD4-mediated ubiquitination of chondroitin sulfate synthase 1 (CHSY1), which promotes CRC proliferation by mediating the nuclear factor-κB (NF-κB) and caspase-3/7 signaling pathways [92]. A study showed that NEDD4 targets the transcription factor YY1 (YY1) for its ubiquitination and degradation, resulting in a decrease in the ratio of full-length LEF 1 (FL-LEF 1)/dominant-negative LEF 1 (DN-LEF 1) transcripts, which then inhibits the activation of WNT signaling and the growth of intestinal tumors in Apc+/min mice [64]. Notably, Jarred P. Tanksley et al. investigated the expression of the NEDD4 family based on microarray analysis of 250 tumor samples from CRC patients at different stages, six adenoma samples, and 10 adjacent normal samples, and found that the most pronounced upregulation and downregulation was observed in NEDD4 and NEDD4L, respectively. Based on such results, it was hypothesized that NEDD4L is a tumor suppressor of CRC and that NEDD4L inhibits classical WNT signaling by targeting disheveled segment polarity protein 2 (DVL 2) for proteasomal degradation so as to inhibit tumor proliferation [74]. Further, it has been demonstrated that copper is associated with colon cancer by enhancing the function of autophagy ULK kinase [95,96]. Jianping Guo et al. concluded that copper activated protein kinase B (PK B) (also known as AKT) in a copper transporter protein (CTR1)-dependent manner and bound to phosphatidylinositol-dependent 3-phosphate kinase 1) to promote the activation of AKT in a phosphatidylinositol 3-kinase (PI3K)-dependent manner, thereby contributing to the development of cancer. Interestingly, the authors also found that NEDD4L exerted its tumor-suppressive effect by targeting CTR to mediate its ubiquitination degradation so as to inhibit the CTR1-AKT signaling pathway. Thus, the copper transporter protein (CTR 1)–copper oncogenicity axis may be a new pharmacological target for the treatment of overactive AKT-driven colon cancer [62,95]. Nonetheless, further in-depth studies are warranted to accurately clarify the roles of NEDD4 and NEDD4L in CRC and the mechanisms of their occurrence in CRC, so that more precise targeted drugs can be developed to improve the therapeutic efficacy for CRC patients (Figure 4) (Table 1).

#### 2.1.4. NEDD4/NEDD4L and Inflammatory Bowel Disease

Inflammatory bowel disease (IBD) is a chronic, progressive immune-mediated inflammatory disease of the gastrointestinal tract, with the main forms including Crohn’s disease (CD) and ulcerative colitis (UC) [128,129]. CD and UC can both progress to CRC, making the pathogenesis of IBD a long-standing research topic [96,129]. It has been demonstrated that NEDD4 restricts T cell function and affects T cell differentiation, and the activators of NEDD4, Ndfip1, and Ndfip2 together restrict the aggregation and function of effector T cells. Meanwhile, a deficiency of T cells in Ndfip1/Ndfip2 can lead to the exacerbation of colitis [130]. Overall, knowledge of the roles of NEDD4 and NEDD4L in IBD is still limited, and in-depth studies on their mechanisms are needed to improve the treatment outcomes of patients with IBD. 

#### 2.1.5. NEDD4/NEDD4L and Diarrhea

Diarrhea is a complex disease, and gastrointestinal water and electrolyte disorders are common causes of diarrhea [131,132]. Han et al. found that metformin might lead to diarrhea by increasing intestinal water loss through inhibition of Na+/H+ exchanger 3 (NHE3, SLC9A3)-dependent fluid absorption; however, NEDD4L knockdown was able to attenuate metformin-induced diarrhea, suggesting that the inhibitory effect of metformin on NHE3 is mediated by ubiquitination of NEDD4L by NHE 3 and that it is the indirect phosphorylation of NEDD4L by 5′-AMP-activated protein kinase (AMPK) that is presumed to promote NH3 activity [97] (Table 1).

### 2.2. NEDD4/NEDDL and Liver Diseases

#### 2.2.1. NEDD4/NEDDL and Liver Cancer

**Figure 5:**
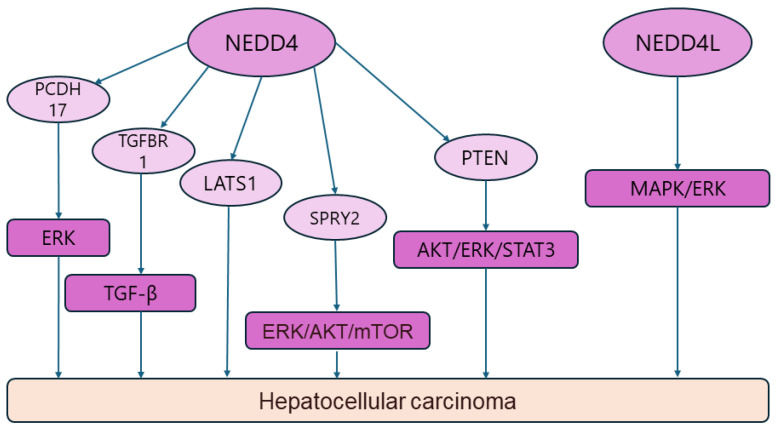
NDDD4/NDDD4L signaling pathway in hepatocellular carcinoma.

Liver cancer is a common malignant tumor and ranks as the sixth most common malignant tumor and the fourth leading cause of cancer-related deaths in the world [133,134,135]. Hepatocellular carcinoma (HCC), a common form of liver cancer, has a complex etiology and its exact pathogenesis has not yet been elucidated. The pathogenesis of HCC is a multifactorial, multistep, and complex process, which is influenced by both environmental and dietary factors [133,136,137]. In the current basic and clinical studies, increasing evidence has suggested that ubiquitination is closely related to HCC, in which the NEDD4 family plays a key role. Susie A. Lee et al. found that the combined effect of loss of activity in sprouty RTK Signaling Antagonist 2 (SPRY2) and overexpression of c-Met could lead to activation of the ERK and AKT/mTOR pathways to induce cell proliferation and angiogenesis, thereby promoting hepatocarcinogenesis. Moreover, it was reported that all HCC cases upregulated by NEDD4 showed low SPRY2 protein levels, suggesting that NEDD4 may mediate the degradation of SPRY2 and be involved in a range of biological processes such as HCC occurrence and metastasis [98]. Xiaofeng Hang and colleagues examined the expression of NEDD4 in 219 HCC tissues. Their results indicated that NEDD4 depletion inhibited the proliferation, migration, and invasion of HCC cells and induced cell cycle arrest in the S-phase, whereas NEDD4 silencing inhibited cell proliferation and altered the assembly of the cytoskeleton of human HCC cells (Huh-7). Therefore, it was hypothesized that NEDD4 interacts directly with PTEN to affect the AKT, ERK1/2, and STAT3 pathways, and thus plays a role in hepatocarcinogenesis and development [138]. It has also been reported that NEDD4 can negatively regulate large tumor suppressor kinase 1 (LATS1) in HCC cells, and that NEDD4 may be involved in the development of HCC by targeting LATS1 for ubiquitination degradation, leading to a decrease in the expression level of LATS1. For this reason, targeting the NEDD4-LATS1 signaling pathway may be a potential therapeutic option for HCC [99]. Recently, Yan Zhou et al. found that craniofacial developmental protein 1 (CFDP1) was highly expressed in HCC tissues and cell lines, and could synergize with NEDD4 to promote the growth and migration of HCC cells via the PTEN/PI3K/AKT pathway. In the meantime, the authors also reported that NEDD4 overexpression enhanced the migration, invasion, and apoptosis of human hepatocellular carcinoma cells (Hep3B cell line) with downregulation of CFDP1, which means that CFDP1 is involved in HCC tumorigenesis and development via the NEDD4-mediated PTEN/PI3K/AKT pathway. These results provide new therapeutic targets for HCC [100]. In another study, Marina Maria Bellet et al. concluded that the guanylyl cyclase domain containing 1 (GUCD1) was strongly associated with liver regeneration and highly expressed in the livers of patients with HCC, and that NEDD4 targeted GUCD1 to mediate its ubiquitination and degradation [139]. Zhaowei Qu et al. conducted a comprehensive bioinformatics study on the potential molecular mechanisms of TGF-β activation in HCC cells, and found that TGF-β may affect the biological processes of HCC cells via regulatory pathways such as SMAD2/SMAD3-NEDD4 and HNF4A-CUL4 B/NEDD4, which provides a new direction for further research on HCC [140]. Zhiyi Liu et al. reported that NEDD4 could regulate the ERK signaling pathway by inducing the ubiquitination degradation of protocadherin 17 (PCDH17), which would then promote the proliferation of HCC cells [101]. Surprisingly, another study revealed that the expression of NEDD4L was downregulated in HCC tissues compared with paraneoplastic tissues, and by analyzing the relationship between NEDD4L and the clinicopathological features of HCC patients, NEDD4L was shown to exert a tumor suppressor effect in HCC by triggering MAPK/ERK-mediated apoptosis. It was therefore hypothesized that NEDD4L is a potential prognostic biomarker and a therapeutic target for HCC [103]. Radiofrequency ablation (RFA), a thermal ablation technique, is one of the effective treatments for early-stage liver cancer [141,142]. However, patients treated with RFA may encounter a condition known as “insufficient ablation”. Kai Li et al. observed that NEDD4 was upregulated in tissues with insufficient HCC ablation and promoted HCC cell migration, suggesting that NEDD4 mediates tumor progression by enhancing TGF-β signaling through direct binding to the TGF-β type I receptor (TGFBR 1) and the formation of a K27-conjugated ubiquitin chain at lysine 391 (i.e., inadequate ablation induced HCC progression owing to the upregulation of NEDD4). This provides a new therapeutic target for HCC [102]. Hexu Han et al. found that NEDD4 binding protein 3 (N4BP3) was highly expressed in HCC, and the C-terminus of N4BP3 interacted with lysine acetyltransferase 2B (KAT2B) to regulate the distribution of the promoter of acetyl-histone H3 (Lys 27) (H3K27 ac) and increase the expression of the signal transducer and activator of transcription 3 (STAT3), thereby increasing the expression and secretion of vascular endothelial growth factor A (VEGFA) to promote angiogenesis in HCC. Furthermore, the efficacy of sorafenib was enhanced, providing a new theoretical basis for the selection of therapeutic targets in HCC [143]. Nonetheless, the exact roles of NEDD 4 and NEDD4L in HCC need to be further investigated to guide future drug research directions and identify effective diagnostic and prognostic markers for HCC to improve patients’ survival (Figure 5) (Table 1).

#### 2.2.2. NEDD4/NEDD4L and Liver Injury

Liver injury generally refers to damage to the liver due to one or more causes, including rupture of the liver by trauma and various liver diseases such as viral hepatitis, drug-induced hepatitis, alcoholic hepatitis, non-alcoholic fatty liver disease, and autoimmune liver disease [144,145,146,147]. Caffeine has been reported to be associated with acute liver injury. Xing-Wang Hu et al. found that caffeine might induce the expression of NEDD4L and degrade glucose-regulated protein 78 (GRP78) through ubiquitination of the NEDD4L lysine at position 324, thereby inhibiting apoptosis and endoplasmic reticulum stress (ERS) to exert its hepatoprotective effect on acute liver injury [106]. Fatty liver is generally categorized into two main groups: alcoholic fatty liver and non-alcoholic fatty liver [148,149]. Among them, non-alcoholic fatty liver disease (NAFLD) is the most common form of liver disease, and non-alcoholic steatohepatitis (NASH) is a progressive form of NAFLD that may eventually lead to cirrhosis of the liver [150,151,152]. Chunchi Yan and colleagues observed that NEDD4 expression was increased in goose fatty liver, which mediated the ubiquitination pathway degradation of the PTEN and IGF 1R proteins to protect against severe steatosis and liver fibrosis, as well as other related injuries. Therefore, goose fatty liver can be used as a special model to study NAFLD and to provide new therapeutic ideas for the development of non-alcoholic steatohepatitis [104]. Qian Guo et al. found that the thioredoxin-interacting protein (TXNIP) aggregated abnormally in the livers of NASH mice, and that the level of TXNIP protein was positively correlated with that of the CCAAT/enhancer-binding protein homologous protein (CHOP). The results indicate that reduction of NEDD4L resulted in TXNIP accumulation in the liver due to impaired ubiquitination degradation, suggesting that TXNIP knockdown can inhibit the expression of CHOP and its downstream apoptotic pathway, therefore exerting an improving effect on NASH by reducing apoptosis, inflammation, and fibrosis in the liver [105]. A recent study found that β-glucosylceramide (β-GluCer)-activated macrophages in hepatic macrophages induced macrophage-inducible C-type lectins (Mincle/Clec4e) to exacerbate hepatocyte cell death and alcohol-associated liver disease by mediating the production and release of interleukin 1β-containing extracellular vesicles (sEVs) via non-cleaved gasdermin D (GSDMD). In this pathway, the full-length GSDMD is accompanied by CDC37/HSP90, which recruits NEDD4 to mediate the polyubiquitylation of pro-IL-1β, thus playing an important role in liver injury [107] (Table 1).

#### 2.2.3. NEDD4/NEDD4L and Liver Fibrosis

Liver fibrosis occurs in the majority of patients with chronic liver diseases. Although the mechanisms of liver fibrosis have been well studied, the current understanding is far from sufficient; as a consequence, there are still limited therapeutic options for the treatment of liver fibrosis [153,154]. Recently, Yu Song et al. found that TGF-β1 could induce NEDD4 family-interacting protein 1 (Ndfip1) to promote the degradation of TrkB via UPS, thereby promoting the progression of liver fibrosis. Thus, TrkB is expected to be a novel therapeutic target for liver fibrosis, which opens up a new way of thinking about the treatment of liver fibrosis [155].

#### 2.2.4. NEDD4/NEDD4L and Portal Hypertension

Portal hypertension is a syndrome caused by a persistent increase in portal venous pressure, mostly consisting of a group of clinical syndromes resulting from increased pressure in the portal venous system caused by cirrhosis [156,157]. Gang Dong et al. investigated the effect of mechanical stretch on endothelial cells (ECs) by simulating portal hypertension using an elastic silicone chamber, and found that the mechanical stretch reduced the expression of peroxisome proliferator-activated receptor γ (PPARγ) in ECs by increasing the NEDD4-mediated ubiquitinated degradation of PPARγ, suggesting that PPARγ may be a novel therapeutic target for portal hypertension [108,157] (Table 1).

### 2.3. NEDD4/NEDD4L and Biliary Diseases

#### 2.3.1. NEDD4/NEDD4L and Gallbladder Cancer

Gallbladder cancer (GBC) is the most common cancer of the biliary tract [158]. The majority of patients with GBC have no obvious symptoms in the early stages, but the prognosis is poor after late diagnosis [159]. The pathogenesis of GBC has not been fully clarified, and it is currently thought to be associated with environmental and genetic factors [158,160]. Tamotsu Takeuchi et al. found that NEDD4L was overexpressed in GBC cells by upregulating matrix metalloproteinase-1 (MMP-1) and matrix metalloproteinase-13 (MMP-13), which increased the invasive activity of GBC cells, suggesting that NEDD4L may be a potential invasive factor in GBC. Therefore, NEDD4L could be targeted as a research direction for new drugs to improve the survival of patients with GBC [109]; however, the underlying mechanisms of NEDD4 and NEDD4L in this disease still need to be further elucidated (Table 1).

#### 2.3.2. NEDD4/NEDD4L and Cholangiocarcinoma

**Figure 6:**
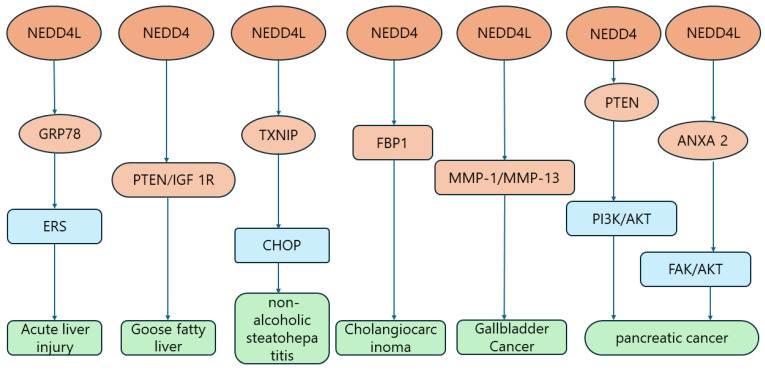
NDDD4/NDDD4L signaling pathway in liver injury/gallbladder cancer/bile duct cancer/pancreatic cancer.

Cholangiocarcinoma (CCA) is a highly heterogeneous malignant tumor of the hepatobiliary system [161], and the overall morbidity and mortality of CCA are still increasing in the global context [162]. Most CCA cases have no clear etiology and have been mainly associated with gallstones, chronic cholecystitis, and cholestasis, and the patients are often diagnosed at an advanced stage of cancer with a poor prognosis [163,164]. Therefore, more research effort is needed to tap into various markers of cancer to increase the rate of early diagnosis and to improve the patients’ treatment outcomes and prognosis. Currently, it has been found that the expression of nuclear factor of activated T cells 2 (NFATC2) is upregulated in CCA tissues and cells, and NEDD4 is also upregulated in human CCA samples, suggesting a positive correlation between the two. The experimental results indicate that NFATC2 can be enriched in the promoter region of NEDD4 to promote its expression. In addition, NEDD4 targets fructose-bisphosphatase 1 (FBP 1) and inhibits FBP1 expression by ubiquitination, i.e., NFATC2 promotes the malignant progression of CCA via the NEDD4/FBP1 axis. Thus, targeting NFATC2 has great therapeutic potential for cholangiocarcinoma [110] (Figure 6) (Table 1).

#### 2.3.3. NEDD4/NEDD4L and Bile Duct Malformations

Autosomal recessive polycystic kidney disease (ARPKD) is an important childhood nephropathy with a predominant clinical phenotype manifesting in the kidneys with collecting duct dilatation, systemic hypertension, and progressive renal insufficiency, as well as in the liver with biliary dysplasia, portal fibrosis, and portal hypertension [165,166]. It has been revealed that the fibrocystin/multicatheterin complex (FPC) interacts with the Nedd4 family-interacting protein 2 (Ndfip 2), while disruption of Pkhd1 function alters the subcellular localization and function of these ligases, leading to increased activity of TGF-β in the bile duct epithelial cells and of ENaC in the collecting duct cells. Consequently, the number of irregularly shaped and dilated bile ducts is increased, accompanied by varying degrees of associated fibrosis [167] (Table 1).

### 2.4. NEDD4/NEDD4L and Pancreatic Diseases

NEDD4/NEDD4L and Pancreatic Cancer

Pancreatic cancer (PC), with the title of “king of cancer”, is a common malignant cancer of the digestive tract and is a main cause of cancer-related deaths worldwide. Pancreatic cancer has atypical symptoms, is difficult to diagnose and treat, and has one of the worst prognoses of all malignant diseases [168,169,170]. Some studies have previously reported that nicotine can promote the development of pancreatic ductal adenocarcinoma (PDAC) cells via the microRNA-155-5p/NEDD4 family-interacting protein 1 (NDFIP1) axis [171]. Adam E. Frampton et al. identified three miRNAs (MIR21, MIR23A, and MIR27A) that can promote the progression of PDAC by targeting the triad of NEDD4L, programmed cell death factor 4 (PDCD4), and btg anti-proliferation factor 2 (BTG2) [172]. Jie Wang et al. found that LINC00941 competitively bound to membrane-associated protein A2 (ANXA 2) to reduce the binding of ANXA 2 to NEDD4L, thereby inhibiting the degradation of ANXA 2, enhancing the stability of ANXA 2, and subsequently activating FAK/AKT signaling to promote the proliferation and metastasis of PC cells. It was therefore hypothesized that LINC 00941 may be regarded as a new target for PC treatment [111]. Min Weng et al. reported that NEDD4 might affect the PTEN/PI3K/AKT signaling pathway in PDAC cells by negatively regulating PTEN levels in PDAC cells, possibly promoting cancerous processes such as the proliferation, migration, and invasion of PDAC cells [113]. Another study reported that curcumin reduced the migration and invasive ability of PC cells by inhibiting NEDD4, which reconfirms that NEDD4 is directly related to the occurrence and progression of PDAC, and that NEDD4 may be a potential therapeutic target for PDAC [173]. In addition, gemcitabine is a baseline drug for PC treatment. Recent studies have demonstrated that knocking down fat mass and obesity-related proteins (FTOs) can reduce NEDD4 expression and therefore significantly increase the level of PTEN expression, with a consequent effect on the PI3K/AKT pathway, which results in PC cells being resistant to gemcitabine, leading to a poor prognosis. Ubiquitin-specific peptidase 7 (USP7) can de-ubiquitinate FTO to increase FTO protein expression and NEDD4 mRNA stability in a YTH N6-methyladenosine RNA-binding protein F2 (YTHDF2)-dependent manner, which then induces proliferation and gemcitabine resistance in PDAC cells. Thus, targeting FTO may also be a novel therapeutic strategy for the treatment of PDAC patients [112,174]. The mortality rate of PC is extremely high, urging researchers to study its pathogenesis more comprehensively in order to improve treatment efficacy and prolong the survival of patients (Table 1). 

## 3. Conclusions and Prospects

Ubiquitination is involved in most cellular activities, and E3 ligases play a key regulator role in various cellular processes. Specifically, NEDD4 and NEDD4L, as the major E3 ligases of the NEDD4 family, are critical in a variety of pathophysiological processes in digestive diseases and are involved in disease development by mediating the ubiquitination degradation of PTEN, c-Myc, and P21. Therefore, it is of great significance to deeply investigate the various regulatory mechanisms and functions of NEDD4 and NEDD4L in the digestive system, to provide new understanding and insights into the occurrence and development of digestive diseases, and to enlighten new directions for drug development against digestive diseases.

## Figures and Tables

**Table 1:** Role of NDDD4/NDDD4L in digestive system disorders.

Disease Classification	NEDD4/NEDD4L	Substrate	Role	Bibliography
Esophageal cancer	NEDD4L ↓	c-Myc	Inhibit cell viability, cell cycle progression, and glutamine metabolism in ESCC	[81]
Gastric cancer	NEDD4 ↑	CNrasGEF	Promote gastric cancer invasion and metastasis	[82]
		PTEN	Promote IGF1 signalling-pathway-driven GC cell growth	[83,84]
		SHIP 2	Promote GC cell proliferation	[85]
	NEDD4 ↓	PDLIM 7	Lead to resistance of GC cells to 5-Fu	[86,87]
	NEDD4L ↓	RDX	Inhibition of GC cell proliferation and migration	[88]
Colorectal cancer	NEDD4 ↑	PTEN	Promotion of colorectal cancer	[89]
		FOXA1	Facilitate the development of CC	[90]
		c-myc	Inhibition of CRC cell proliferation and metastasis	[91]
		CHSY1	Promote proliferation of colorectal cancer cells	[92,93]
	NEDD4 ↓	P21	Inhibition of colorectal tumor proliferation	[94]
	NEDD4 (Unknown)	YY1	Inhibition of intestinal tumor growth in Apc+/min mice	[64]
	NEDD4L ↓	DVL 2	Inhibition of tumor proliferation	[95,96]
	NEDD4L (Unknown)	CTR	Tumor suppressant	[62,95,96]
Diarrhea	NEDD4L ↓	Unknown	Reduction of metformin-induced diarrhea	[97]
HCC	NEDD4 ↑	SPRY2 (likelihood)	Involved in HCC genesis, metastasis, etc.	[98]
		LATS1	Involved in the development of HCC	[99]
		PTEN	Involved in HCC tumorigenesis and progression	[100]
		PCDH17	Promote the proliferation of hepatocellular carcinoma cells	[101]
		TGFBR 1	Promote progress on HCC	[102]
	NEDD4 (Unknown)	PTEN (likelihood)	Involved in the development of liver cancer	[98]
	NEDD4L ↓	Unknown	Tumor suppressant	[103]
Non-alcoholic steatohepatitis	NEDD4 ↑	PTEN	Protect fatty liver from severe steatosis and associated damage such as liver fibrosis	[104]
	NEDD4L (Unknown)	TXNIP	Reduce liver apoptosis, inflammation, and fibrosis to improve NASH	[105]
Acute liver injury	NEDD4L ↓	GRP78	Hepatoprotective effect	[106]
Alcohol-related liver disease	NEDD4 (Unknown)	pro-IL-1β	Exacerbation of liver cell death and alcohol-related liver disease	[107]
Portal hypertension	NEDD4 ↑	PPARγ	Reduce PPARγ expression in endothelial cells	[108]
Gallbladder Cancer	NEDD4L ↑	MMP-1/MMP-13	Increase invasiveness of gallbladder cancer	[109]
Cholangiocarcinoma	NEDD4 ↑	FBP 1	Promote malignant progression of CCA	[110]
PC	NEDD4L ↓	ANXA 2	Promote PC proliferation and metastasis	[111]
	NEDD4 ↑	PTEN (likelihood)	Promote cancerous processes such as proliferation, migration, and invasion of PDAC cells	[112,113]

## References

[1] Çetin G., Klafack S., Studencka-Turski M., Krüger E., Ebstein F. (2023). The Ubiquitin–Proteasome System in Immune Cells. Biomolecules.

[2] Finley D. (2009). Recognition and Processing of Ubiquitin-Protein Conjugates by the Proteasome. Annu. Rev. Biochem..

[3] Ciechanover A. (2006). The ubiquitin proteolytic system: From a vague idea, through basic mechanisms, and onto human diseases and drug targeting. Neurology.

[4] Park J., Cho J., Song E.J. (2020). Ubiquitin-proteasome system (UPS) as a target for anticancer treatment. Arch. Pharmacal Res..

[5] Gierisch M.E., Giovannucci T.A., Dantuma N.P. (2014). Reporter-Based Screens for the Ubiquitin/Proteasome System. Front. Chem..

[6] Shaid S., Brandts C.H., Serve H., Dikic I. (2013). Ubiquitination and selective autophagy. Cell Death Differ..

[7] Yau R., Rape M. (2016). The increasing complexity of the ubiquitin code. Nat. Cell Biol..

[8] Morimoto D., Walinda E., Sugase K., Shirakawa M. (2017). Biological and Physicochemical Functions of Ubiquitylation Revealed by Synthetic Chemistry Approaches. Int. J. Mol. Sci..

[9] Chen R.-H., Chen Y.-H., Huang T.-Y. (2019). Ubiquitin-mediated regulation of autophagy. J. Biomed. Sci..

[10] Li M., Sun G., Wang P., Wang W., Cao K., Song C., Sun Y., Zhang Y., Zhang N. (2022). Research progress of Nedd4L in cardiovascular diseases. Cell Death Discov..

[11] Swatek K.N., Komander D. (2016). Ubiquitin modifications. Cell Res..

[12] Wang J., Maldonado M.A. (2006). The Ubiquitin-Proteasome System and Its Role in Inflammatory and Autoimmune Diseases. Cell. Mol. Immunol..

[13] Song L., Luo Z.-Q. (2019). Post-translational regulation of ubiquitin signaling. J. Cell Biol..

[14] Squair D.R., Virdee S. (2022). A new dawn beyond lysine ubiquitination. Nat. Chem. Biol..

[15] Balbinott N., Margis R. (2023). The many faces of lysine acylation in proteins: Phytohormones as unexplored substrates. Plant Sci..

[16] Gui W., Davidson G.A., Zhuang Z. (2021). Chemical methods for protein site-specific ubiquitination. RSC Chem. Biol..

[17] Rahman S., Wolberger C. (2024). Breaking the K48-chain: Linking ubiquitin beyond protein degradation. Nat. Struct. Mol. Biol..

[18] Xu M., Skaug B., Zeng W., Chen Z.J. (2009). A ubiquitin replacement strategy in human cells reveals distinct mechanisms of IKK activation by TNFalpha and IL-1beta. Mol. Cell.

[19] Deng L., Wang C., Spencer E., Yang L., Braun A., You J., Slaughter C., Pickart C., Chen Z.J. (2000). Activation of the IkappaB kinase complex by TRAF6 requires a dimeric ubiquitin-conjugating enzyme complex and a unique polyubiquitin chain. Cell.

[20] Stewart G.S., Panier S., Townsend K., Al-Hakim A.K., Kolas N.K., Miller E.S., Nakada S., Ylanko J., Olivarius S., Mendez M. (2009). The RIDDLE syndrome protein mediates a ubiquitin-dependent signaling cascade at sites of DNA damage. Cell.

[21] Hoege C., Pfander B., Moldovan G.-L., Pyrowolakis G., Jentsch S. (2002). RAD6-dependent DNA repair is linked to modification of PCNA by ubiquitin and SUMO. Nature.

[22] Gack M.U., Shin Y.C., Joo C.-H., Urano T., Liang C., Sun L., Takeuchi O., Akira S., Chen Z., Inoue S. (2007). TRIM25 RING-finger E3 ubiquitin ligase is essential for RIG-I-mediated antiviral activity. Nature.

[23] Gao P., Ma X., Yuan M., Yi Y., Liu G., Wen M., Jiang W., Ji R., Zhu L., Tang Z. (2021). E3 ligase Nedd4l promotes antiviral innate immunity by catalyzing K29-linked cysteine ubiquitination of TRAF3. Nat. Commun..

[24] Madiraju C., Novack J.P., Reed J.C., Matsuzawa S., Sheka A.C., Adeyi O., Thompson J., Hameed B., Crawford P.A., Ikramuddin S. (2022). K63 ubiquitination in immune signaling. JAMA.

[25] Ohtake F., Tsuchiya H., Saeki Y., Tanaka K. (2018). K63 ubiquitylation triggers proteasomal degradation by seeding branched ubiquitin chains. Proc. Natl. Acad. Sci. USA.

[26] Tannapfel A., Denk H., Dienes H.-P., Langner C., Schirmacher P., Trauner M., Flott-Rahmel B. (2021). Beyond K48 and K63: Non-canonical protein ubiquitination. Cell. Mol. Biol. Lett..

[27] Grice G.L., Lobb I.T., Weekes M.P., Gygi S.P., Antrobus R., Nathan J.A. (2015). The Proteasome Distinguishes between Heterotypic and Homotypic Lysine-11-Linked Polyubiquitin Chains. Cell Rep..

[28] Ordureau A., Heo J.-M., Duda D.M., Paulo J.A., Olszewski J.L., Yanishevski D., Rinehart J., Schulman B.A., Harper J.W. (2015). Defining roles of PARKIN and ubiquitin phosphorylation by PINK1 in mitochondrial quality control using a ubiquitin replacement strategy. Proc. Natl. Acad. Sci. USA.

[29] Cassidy K.B., Bang S., Kurokawa M., Gerber S.A. (2020). Direct regulation of Chk1 protein stability by E3 ubiquitin ligase HUWE1. FEBS J..

[30] Gatti M., Pinato S., Maiolica A., Rocchio F., Prato M.G., Aebersold R., Penengo L. (2015). RNF168 promotes noncanonical K27 ubiquitination to signal DNA damage. Cell Rep..

[31] Liu C., Liu W., Ye Y., Li W. (2017). Ufd2p synthesizes branched ubiquitin chains to promote the degradation of substrates modified with atypical chains. Nat. Commun..

[32] Chastagner P., Israël A., Brou C. (2006). Itch/AIP4 mediates Deltex degradation through the formation of K29-linked polyubiquitin chains. EMBO Rep..

[33] Nibe Y., Oshima S., Kobayashi M., Maeyashiki C., Matsuzawa Y., Otsubo K., Matsuda H., Aonuma E., Nemoto Y., Nagaishi T. (2018). Novel polyubiquitin imaging system, PolyUb-FC, reveals that K33-linked polyubiquitin is recruited by SQSTM1/p62. Autophagy.

[34] Yuan W.-C., Lee Y.-R., Lin S.-Y., Chang L.-Y., Tan Y.P., Hung C.-C., Kuo J.-C., Liu C.-H., Lin M.-Y., Xu M. (2014). K33-Linked Polyubiquitination of Coronin 7 by Cul3-KLHL20 Ubiquitin E3 Ligase Regulates Protein Trafficking. Mol. Cell.

[35] Dagar G., Kumar R., Yadav K.K., Singh M., Pandita T.K. (2023). Ubiquitination and deubiquitination: Implications on cancer therapy. Biochim. Biophys. Acta (BBA) Gene Regul. Mech..

[36] Xu K., Chu Y., Liu Q., Fan W., He H., Huang F. (2022). NEDD4 E3 Ligases: Functions and Mechanisms in Bone and Tooth. Int. J. Mol. Sci..

[37] Buetow L., Huang D.T. (2016). Structural insights into the catalysis and regulation of E3 ubiquitin ligases. Nat. Rev. Mol. Cell Biol..

[38] Berndsen C.E., Wolberger C. (2014). New insights into ubiquitin E3 ligase mechanism. Nat. Struct. Mol. Biol..

[39] d’Azzo A., Bongiovanni A., Nastasi T. (2005). E3 Ubiquitin Ligases as Regulators of Membrane Protein Trafficking and Degradation. Traffic.

[40] Jayaprakash S., Hegde M., BharathwajChetty B., Girisa S., Alqahtani M.S., Abbas M., Sethi G., Kunnumakkara A.B. (2022). Unraveling the Potential Role of NEDD4-like E3 Ligases in Cancer. Int. J. Mol. Sci..

[41] Wang Y., Argiles-Castillo D., Kane E.I., Zhou A., Spratt D.E. (2020). HECT E3 ubiquitin ligases—Emerging insights into their biological roles and disease relevance. J. Cell Sci..

[42] Sun A., Chen Y., Tian X., Lin Q. (2014). The Role of HECT E3 Ubiquitin Ligases in Colorectal Cancer. Biomedicines.

[43] Zheng N., Shabek N. (2017). Ubiquitin Ligases: Structure, Function, and Regulation. Annu. Rev. Biochem..

[44] Qian H., Zhang Y., Wu B., Wu S., You S., Zhang N., Sun Y., Arasaradnam R.P., Brown S., Forbes A. (2020). Structure and function of HECT E3 ubiquitin ligases and their role in oxidative stress. J. Transl. Int. Med..

[45] Wang D., Ma L., Wang B., Liu J., Wei W. (2017). E3 ubiquitin ligases in cancer and implications for therapies. Cancer Metastasis Rev..

[46] Song M.S., Pandolfi P.P. (2022). The HECT family of E3 ubiquitin ligases and PTEN. Semin. Cancer Biol..

[47] Rotin D., Kumar S. (2009). Physiological functions of the HECT family of ubiquitin ligases. Nat. Rev. Mol. Cell Biol..

[48] Zhang Y., Qian H., Wu B., You S., Wu S., Lu S., Wang P., Cao L., Zhang N., Sun Y. (2020). E3 Ubiquitin ligase NEDD4 family-regulatory network in cardiovascular disease. Int. J. Biol. Sci..

[49] Tian X., Chen Y., Peng Z., Lin Q., Sun A. (2023). NEDD4 E3 ubiquitin ligases: Promising biomarkers and therapeutic targets for cancer. Biochem. Pharmacol..

[50] Persaud A., Alberts P., Amsen E.M., Xiong X., Wasmuth J., Saadon Z., Fladd C., Parkinson J., Rotin D. (2009). Comparison of substrate specificity of the ubiquitin ligases Nedd4 and Nedd4-2 using proteome arrays. Mol. Syst. Biol..

[51] Ingham R.J., Gish G., Pawson T. (2004). The Nedd4 family of E3 ubiquitin ligases: Functional diversity within a common modular architecture. Oncogene.

[52] Pohl P., Joshi R., Petrvalska O., Obsil T., Obsilova V. (2021). 14-3-3-protein regulates Nedd4-2 by modulating interactions between HECT and WW domains. Commun. Biol..

[53] Spagnol G., Kieken F., Kopanic J.L., Li H., Zach S., Stauch K.L., Grosely R., Sorgen P.L. (2016). Structural Studies of the Nedd4 WW Domains and Their Selectivity for the Connexin43 (Cx43) Carboxyl Terminus. J. Biol. Chem..

[54] Jiang H., Thomas S.N., Chen Z., Chiang C.Y., Cole P.A. (2019). Comparative analysis of the catalytic regulation of NEDD4-1 and WWP2 ubiquitin ligases. J. Biol. Chem..

[55] Wan L., Liu T., Hong Z., Pan Y., Sizemore S.T., Zhang J., Ma Z. (2019). NEDD4 expression is associated with breast cancer progression and is predictive of a poor prognosis. Breast Cancer Res..

[56] Ye X., Wang L., Shang B., Wang Z., Wei W., Gierisch M.E., Giovannucci T.A., Dantuma N.P. (2014). NEDD4: A Promising Target for Cancer Therapy. Curr. Cancer Drug Targets.

[57] Sun L., Amraei R., Rahimi N. (2021). NEDD4 regulates ubiquitination and stability of the cell adhesion molecule IGPR-1 via lysosomal pathway. J. Biomed. Sci..

[58] Xu J., Sheng Z., Li F., Wang S., Yuan Y., Wang M., Yu Z. (2019). NEDD4 protects vascular endothelial cells against Angiotensin II-induced cell death via enhancement of XPO1-mediated nuclear export. Exp. Cell Res..

[59] Eide P.W., Cekaite L., Danielsen S.A., Eilertsen I.A., Kjenseth A., Fykerud T.A., Ågesen T.H., Bruun J., Rivedal E., Lothe R.A. (2013). NEDD4 is overexpressed in colorectal cancer and promotes colonic cell growth independently of the PI3K/PTEN/AKT pathway. Cell. Signal..

[60] Jing W., Wang G., Cui Z., Xiong G., Jiang X., Li Y., Li W., Han B., Chen S., Shi B. (2022). FGFR3 Destabilizes PD-L1 via NEDD4 to Control T-cell-Mediated Bladder Cancer Immune Surveillance. Cancer Res..

[61] Zhang L., Qin Y., Wu G., Wang J., Cao J., Wang Y., Wu D., Yang K., Zhao Z., He L. (2020). PRRG4 promotes breast cancer metastasis through the recruitment of NEDD4 and downregulation of Robo1. Oncogene.

[62] Guo J., Cheng J., Zheng N., Zhang X., Dai X., Zhang L., Hu C., Wu X., Jiang Q., Wu D. (2021). Copper Promotes Tumorigenesis by Activating the PDK1-AKT Oncogenic Pathway in a Copper Transporter 1 Dependent Manner. Adv. Sci..

[63] Trotman L.C., Wang X., Alimonti A., Chen Z., Teruya-Feldstein J., Yang H., Pavletich N.P., Carver B.S., Cordon-Cardo C., Erdjument-Bromage H. (2007). Ubiquitination regulates PTEN nuclear import and tumor suppression. Cell.

[64] Lu C., Thoeni C., Connor A., Kawabe H., Gallinger S., Rotin D. (2016). Intestinal knockout of Nedd4 enhances growth of Apcmin tumors. Oncogene.

[65] Zeng T., Wang Q., Fu J., Lin Q., Bi J., Ding W., Qiao Y., Zhang S., Zhao W., Lin H. (2014). Impeded Nedd4-1-mediated Ras degradation underlies Ras-driven tumorigenesis. Cell Rep..

[66] Russo C.J., Melista E., Cui J., DeStefano A.L., Bakris G.L., Manolis A.J., Gavras H., Baldwin C.T. (2005). Association of NEDD4L ubiquitin ligase with essential hypertension. Hypertension.

[67] Kamynina E., Debonneville C., Bens M., Vandewalle A., Staub O. (2001). A novel mouse Nedd4 protein suppresses the activity of the epithelial Na+ channel. FASEB J..

[68] Mohammed M., Ogunlade B., Elgazzaz M., Berdasco C., Lakkappa N., Ghita I., Guidry J.J., Sriramula S., Xu J., Restivo L. (2023). Nedd4-2 up-regulation is associated with ACE2 ubiquitination in hypertension. Cardiovasc. Res..

[69] Persaud A., Alberts P., Hayes M., Guettler S., Clarke I., Sicheri F., Dirks P., Ciruna B., Rotin D. (2011). Nedd4-1 binds and ubiquitylates activated FGFR1 to control its endocytosis and function. EMBO J..

[70] Henshall T.L., Manning J.A., Alfassy O.S., Goel P., Boase N.A., Kawabe H., Kumar S. (2017). Deletion of Nedd4-2 results in progressive kidney disease in mice. Cell Death Differ..

[71] Leitz D.H.W., Duerr J., Mulugeta S., Seyhan Agircan A., Zimmermann S., Kawabe H., Dalpke A.H., Beers M.F., Mall M.A. (2021). Congenital Deletion of Nedd4-2 in Lung Epithelial Cells Causes Progressive Alveolitis and Pulmonary Fibrosis in Neonatal Mice. Int. J. Mol. Sci..

[72] Li S., Ye Q., Wei J., Taleb S.J., Wang H., Zhang Y., Kass D.J., Horowitz J.C., Zhao J., Zhao Y. (2023). Nedd4L suppression in lung fibroblasts facilitates pathogenesis of lung fibrosis. Transl. Res..

[73] Lee D.-E., Yoo J.E., Kim J., Kim S., Kim S., Lee H., Cheong H. (2020). NEDD4L downregulates autophagy and cell growth by modulating ULK1 and a glutamine transporter. Cell Death Dis..

[74] Tanksley J.P., Chen X., Coffey R.J. (2013). NEDD4L is downregulated in colorectal cancer and inhibits canonical WNT signaling. PLoS ONE.

[75] Waters J.K., Reznik S.I. (2022). Update on Management of Squamous Cell Esophageal Cancer. Curr. Oncol. Rep..

[76] An L., Li M., Jia Q. (2014). Mechanisms of radiotherapy resistance and radiosensitization strategies for esophageal squamous cell carcinoma. Mol. Cancer.

[77] Yang T., Hui R., Nouws J., Sauler M., Zeng T., Wu Q. (2022). Untargeted metabolomics analysis of esophageal squamous cell cancer progression. J. Transl. Med..

[78] Codipilly D.C., Wang K.K. (2022). Squamous Cell Carcinoma of the Esophagus. Gastroenterol. Clin. N. Am..

[79] Kelly R.J. (2019). Emerging Multimodality Approaches to Treat Localized Esophageal Cancer. J. Natl. Compr. Cancer Netw..

[80] Xin Z., Liu Q., Ai D., Chen K., Mariamidze E., Sumon M.A., Devnani B., Pihlak R., Zhu H., Zhao K. (2023). Radiotherapy for Advanced Esophageal Cancer: From Palliation to Curation. Curr. Treat. Options Oncol..

[81] Cheng W., Li G., Ye Z., Hu J., Gao L., Jia X., Zhao S., Wang Y., Zhou Q. (2022). NEDD4L inhibits cell viability, cell cycle progression, and glutamine metabolism in esophageal squamous cell carcinoma via ubiquitination of c-Myc. Acta Biochim. Biophys. Sin..

[82] Li D., Xu C.-Y., Cui R.-J., Tang J.-B., Sun H., Yang Z.-K., Bu J.-Y., Lin P., Huang N., Du Y.-D. (2015). DNA methylation inhibitor, decitabine, promotes MGC803 gastric cancer cell migration and invasion via the upregulation of NEDD4-1. Mol. Med. Rep..

[83] Wozniak D.J., Kajdacsy-Balla A., Macias V., Ball-Kell S., Zenner M.L., Bie W., Tyner A.L. (2017). PTEN is a protein phosphatase that targets active PTK6 and inhibits PTK6 oncogenic signaling in prostate cancer. Nat. Commun..

[84] Wang K., Yu Y., Wang W., Jiang Y., Li Y., Jiang X., Qiao Y., Chen L., Zhao X., Liu J. (2023). Targeting the E3 ligase NEDD4 as a novel therapeutic strategy for IGF1 signal pathway-driven gastric cancer. Oncogene.

[85] Xu L., Xiang W., Yang J., Gao J., Wang X., Meng L., Ye K., Zhao X.H., Zhang X.D., Jin L. (2024). PHB2 promotes SHIP2 ubiquitination via the E3 ligase NEDD4 to regulate AKT signaling in gastric cancer. J. Exp. Clin. Cancer Res..

[86] Lu Y., Jin Z., Hou J., Wu X., Yu Z., Yao L., Pan T., Chang X., Yu B., Li J. (2023). Calponin 1 increases cancer-associated fibroblasts-mediated matrix stiffness to promote chemoresistance in gastric cancer. Matrix Biol..

[87] Ouyang S., Li H., Lou L., Huang Q., Zhang Z., Mo J., Li M., Lu J., Zhu K., Chu Y. (2022). Inhibition of STAT3-ferroptosis negative regulatory axis suppresses tumor growth and alleviates chemoresistance in gastric cancer. Redox Biol..

[88] Tang X., Huang J., Jiang Y., Qiu J., Li T., Li W., Chen Z., Huang Z., Yu X., Yang T. (2023). Intercellular adhesion molecule 2 as a novel prospective tumor suppressor induced by ERG promotes ubiquitination-mediated radixin degradation to inhibit gastric cancer tumorigenicity and metastasis. J. Transl. Med..

[89] Kim S.S., Yoo N.J., Jeong E.G., Kim M.S., Lee S.H. (2008). Expression of NEDD-1, a PTEN regulator, in gastric and colorectal carcinomas. APMIS.

[90] Yue M., Yun Z., Li S., Yan G., Kang Z. (2021). NEDD4 triggers FOXA1 ubiquitination and promotes colon cancer progression under microRNA-340-5p suppression and ATF1 upregulation. RNA Biol..

[91] Wang Y.-N., Ruan D.-Y., Wang Z.-X., Yu K., Rong D.-L., Liu Z.-X., Wang F., Hu J.-J., Jin Y., Wu Q.-N. (2022). Targeting the cholesterol-RORα/γ axis inhibits colorectal cancer progression through degrading c-myc. Oncogene.

[92] Zeng L., Qian J., Luo X., Zhou A., Zhang Z., Fang Q. (2018). CHSY1 promoted proliferation and suppressed apoptosis in colorectal cancer through regulation of the NFκB and/or caspase-3/7 signaling pathway. Oncol. Lett..

[93] Yu S., Dai W., Zhao S., Yang Y., Xu Y., Wang J., Deng Q., He J., Shi D. (2023). Function and mechanism of MCM8 in the development and progression of colorectal cancer. J. Transl. Med..

[94] Zhang S., Yu C., Yang X., Hong H., Lu J., Hu W., Hao X., Li S., Aikemu B., Yang G. (2019). N-myc downstream-regulated gene 1 inhibits the proliferation of colorectal cancer through emulative antagonizing NEDD4-mediated ubiquitylation of p21. J. Exp. Clin. Cancer Res..

[95] Tsang T., Posimo J.M., Gudiel A.A., Cicchini M., Feldser D.M., Brady D.C. (2020). Copper is an essential regulator of the autophagic kinases ULK1/2 to drive lung adenocarcinoma. Nat. Cell Biol..

[96] Plevris N., Lees C.W. (2022). Disease Monitoring in Inflammatory Bowel Disease: Evolving Principles and Possibilities. Gastroenterology.

[97] Han Y., Yun C.C. (2022). Metformin Inhibits Na+/H+ Exchanger NHE3 Resulting in Intestinal Water Loss. Front. Physiol..

[98] Lee S.A., Ladu S., Evert M., Dombrowski F., De Murtas V., Chen X., Calvisi D.F. (2010). Synergistic role of Sprouty2 inactivation and c-Met up-regulation in mouse and human hepatocarcinogenesis. Hepatology.

[99] Zheng H., Ke X., Li D., Wang Q., Wang J., Liu X., Deng M., Deng X., Xue Y., Zhu Y. (2018). NEDD4 promotes cell growth and motility in hepatocellular carcinoma. Cell Cycle.

[100] Zhou Y., Qiu J., Liu S., Wang P., Ma D., Zhang G., Cao Y., Hu L., Wang Z., Wu J. (2022). CFDP1 promotes hepatocellular carcinoma progression through activating NEDD4/PTEN/PI3K/AKT signaling pathway. Cancer Med..

[101] Liu Z., Hu Q., Hu B., Cao K., Xu T., Hou T., Cao T., Wang R., Shi H., Zhang B. (2024). Ubiquitin ligase NEDD4 promotes the proliferation of hepatocellular carcinoma cells through targeting PCDH17 protein for ubiquitination and degradation. J. Biol. Chem..

[102] Li K., Niu Y., Yuan Y., Qiu J., Shi Y., Zhong C., Qiu Z., Li K., Lin Z., Huang Z. (2022). Insufficient ablation induces E3-ligase Nedd4 to promote hepatocellular carcinoma progression by tuning TGF-β signaling. Oncogene.

[103] Zhao F., Gong X., Liu A., Lv X., Hu B., Zhang H. (2018). Downregulation of Nedd4L predicts poor prognosis, promotes tumor growth and inhibits MAPK/ERK signal pathway in hepatocellular carcinoma. Biochem. Biophys. Res. Commun..

[104] Yan C., Zhao M., Li S., Liu T., Xu C., Liu L., Geng T., Gong D. (2020). Increase of E3 ubiquitin ligase NEDD4 expression leads to degradation of its target proteins PTEN/IGF1R during the formation of goose fatty liver. J. Anim. Sci..

[105] Guo Q., Xin M., Lu Q., Feng D., Yang V., Peng L.F., Whelan K.A., Hu W., Wu S., Yang X. (2023). A novel NEDD4L-TXNIP-CHOP axis in the pathogenesis of nonalcoholic steatohepatitis. Theranostics.

[106] Hu X.-W., Li X.-M., Wang A.-M., Fu Y.-M., Zhang F.-J., Zeng F., Cao L.-P., Long H., Xiong Y.-H., Xu J. (2022). Caffeine alleviates acute liver injury by inducing the expression of NEDD4L and deceasing GRP78 level via ubiquitination. Inflamm. Res..

[107] Zhang Q., Liu W., Bulek K., Wang H., McMullen M.R., Wu X., Welch N., Zhang R., Dasarathy J., Dasarathy S. (2023). Mincle-GSDMD-mediated release of IL-1β small extracellular vesicles from hepatic macrophages in ethanol-induced liver injury. Hepatol. Commun..

[108] Dong G., Huang X., Xu Y., Chen R., Chen S. (2023). Mechanical stress induced EndoMT in endothelial cells through PPARγ downregulation. Cell Signal.

[109] Takeuchi T., Adachi Y., Nagayama T., Furihata M. (2011). Nedd4L modulates the transcription of metalloproteinase-1 and -13 genes to increase the invasive activity of gallbladder cancer. Int. J. Exp. Pathol..

[110] Zhao W., Zhao J., Li K., Hu Y., Yang D., Tan B., Shi J. (2023). Oncogenic Role of the NFATC2/NEDD4/FBP1 Axis in Cholangiocarcinoma. Lab. Investig..

[111] Wang J., He Z., Liu X., Xu J., Jiang X., Quan G., Jiang J. (2022). LINC00941 promotes pancreatic cancer malignancy by interacting with ANXA2 and suppressing NEDD4L-mediated degradation of ANXA2. Cell Death Dis..

[112] Lin K., Zhou E., Shi T., Zhang S., Zhang J., Zheng Z., Pan Y., Gao W., Yu Y. (2023). m6A eraser FTO impairs gemcitabine resistance in pancreatic cancer through influencing NEDD4 mRNA stability by regulating the PTEN/PI3K/AKT pathway. J. Exp. Clin. Cancer Res..

[113] Weng M., Luo Z.-L., Wu X.-L., Zeng W.-Z. (2017). The E3 ubiquitin ligase NEDD4 is translationally upregulated and facilitates pancreatic cancer. Oncotarget.

[114] Ajani J.A., D’Amico T.A., Bentrem D.J., Chao J., Cooke D., Corvera C., Das P., Enzinger P.C., Enzler T., Fanta P. (2022). Gastric Cancer, Version 2.2022, NCCN Clinical Practice Guidelines in Oncology. J. Natl. Compr. Cancer Netw..

[115] Xia J.Y., Aadam A.A. (2022). Advances in screening and detection of gastric cancer. Clin. Med..

[116] Smyth E.C., Nilsson M., Grabsch H.I., van Grieken N.C., Lordick F. (2020). Gastric cancer. Lancet.

[117] Thrift A.P., El-Serag H.B. (2020). Burden of Gastric Cancer. Clin. Gastroenterol. Hepatol..

[118] Lordick F., Carneiro F., Cascinu S., Fleitas T., Haustermans K., Piessen G., Vogel A., Smyth E.C. (2022). Gastric cancer: ESMO Clinical Practice Guideline for diagnosis, treatment and follow-up. Ann. Oncol..

[119] Wang Y.-Y., Ye Z.-Y., Zhao Z.-S., Tao H.-Q., Li S.-G. (2010). Systems biology approach to identification of biomarkers for metastatic progression in gastric cancer. J. Cancer Res. Clin. Oncol..

[120] Yang Z. (2012). Is NEDD4-1 a negative regulator of phosphatase and tensin homolog in gastric carcinogenesis?. World J. Gastroenterol..

[121] Sun A., Tian X., Chen Y., Yang W., Lin Q. (2023). Emerging roles of the HECT E3 ubiquitin ligases in gastric cancer. Pathol. Oncol. Res..

[122] Sun A., Yu G., Dou X., Yan X., Yang W., Lin Q. (2014). Nedd4-1 is an exceptional prognostic biomarker for gastric cardia adenocarcinoma and functionally associated with metastasis. Mol. Cancer.

[123] Ye H., Shi W., Yang J., Wang L., Jiang X., Zhao H., Qin L., Qin J., Li L., Cai W. (2023). PICH Activates Cyclin A1 Transcription to Drive S-Phase Progression and Chemoresistance in Gastric Cancer. Cancer Res..

[124] Jiang X., Zhang S., Yin Z., Sheng Y., Yan Q., Sun R., Lu M., Zhang Z., Li Y. (2019). The correlation between NEDD4L and HIF-1α levels as a gastric cancer prognostic marker. Int. J. Med. Sci..

[125] Zhao W., Dai S., Yue L., Xu F., Gu J., Dai X., Qian X. (2022). Emerging mechanisms progress of colorectal cancer liver metastasis. Front. Endocrinol..

[126] Dekker E., Tanis P.J., Vleugels J.L.A., Kasi P.M., Wallace M.B. (2019). Colorectal cancer. Lancet.

[127] Biller L.H., Schrag D. (2021). Diagnosis and Treatment of Metastatic Colorectal Cancer: A Review. JAMA.

[128] Zhang Y.-Z. (2014). Inflammatory bowel disease: Pathogenesis. World J. Gastroenterol..

[129] Singh N., Bernstein C.N. (2022). Environmental risk factors for inflammatory bowel disease. United Eur. Gastroenterol. J..

[130] Ramon H.E., Riling C.R., Bradfield J., Yang B., Hakonarson H., Oliver P.M. (2011). The ubiquitin ligase adaptor Ndfip1 regulates T cell-mediated gastrointestinal inflammation and inflammatory bowel disease susceptibility. Mucosal Immunol..

[131] Keely S.J., Barrett K.E. (2022). Intestinal secretory mechanisms and diarrhea. Am. J. Physiol. Gastrointest. Liver Physiol..

[132] Arasaradnam R.P., Brown S., Forbes A., Fox M.R., Hungin P., Kelman L., Major G., O’Connor M., Sanders D.S., Sinha R. (2018). Guidelines for the investigation of chronic diarrhoea in adults: British Society of Gastroenterology, 3rd edition. Gut.

[133] Yang J.D., Hainaut P., Gores G.J., Amadou A., Plymoth A., Roberts L.R. (2019). A global view of hepatocellular carcinoma: Trends, risk, prevention and management. Nat. Rev. Gastroenterol. Hepatol..

[134] Brown Z.J., Tsilimigras D.I., Ruff S.M., Mohseni A., Kamel I.R., Cloyd J.M., Pawlik T.M. (2023). Management of Hepatocellular Carcinoma: A Review. JAMA Surg..

[135] Vogel A., Meyer T., Sapisochin G., Salem R., Saborowski A. (2022). Hepatocellular carcinoma. Lancet.

[136] Llovet J.M., Kelley R.K., Villanueva A., Singal A.G., Pikarsky E., Roayaie S., Lencioni R., Koike K., Zucman-Rossi J., Finn R.S. (2021). Hepatocellular carcinoma. Nat. Rev. Dis. Primers.

[137] Nagaraju G.P., Dariya B., Kasa P., Peela S., El-Rayes B.F. (2022). Epigenetics in hepatocellular carcinoma. Semin. Cancer Biol..

[138] Hang X., Zhu S., Di H., Wu Z., Chu K., Wang J., Xin H., Yu G., Peng H., Miao X. (2016). NEDD4 Depletion Inhibits Hepatocellular Carcinoma Growth via Targeting PTEN. Cell. Physiol. Biochem..

[139] Bellet M.M., Piobbico D., Bartoli D., Castelli M., Pieroni S., Brunacci C., Chiacchiaretta M., Del Sordo R., Fallarino F., Sidoni A. (2014). NEDD4 controls the expression of GUCD1, a protein upregulated in proliferating liver cells. Cell Cycle.

[140] Qu Z., Li D., Xu H., Zhang R., Li B., Sun C., Dong W., Zhang Y. (2016). CUL4B, NEDD4, and UGT1As involve in the TGF-β signalling in hepatocellular carcinoma. Ann. Hepatol..

[141] Deng Q., He M., Fu C., Feng K., Ma K., Zhang L. (2022). Radiofrequency ablation in the treatment of hepatocellular carcinoma. Int. J. Hyperth..

[142] Kim M.-D. (2021). Microwave thermosphere versus radiofrequency ablation for hepatocellular carcinoma: Are we approaching the time to end the debate?. Clin. Mol. Hepatol..

[143] Han H., Zhu W., Lin T., Liu C., Zhai H. (2022). N4BP3 promotes angiogenesis in hepatocellular carcinoma by binding with KAT2B. Cancer Sci..

[144] Yan C., Hu W., Tu J., Li J., Liang Q., Han S. (2023). Pathogenic mechanisms and regulatory factors involved in alcoholic liver disease. J. Transl. Med..

[145] Mays E.T. (1973). Hepatic Trauma. Lancet Psychiatry.

[146] Katarey D., Verma S. (2016). Drug-induced liver injury. Clin. Med..

[147] Devarbhavi H., Asrani S.K., Arab J.P., Nartey Y.A., Pose E., Kamath P.S. (2023). Global burden of liver disease: 2023 update. J. Hepatol..

[148] Sheka A.C., Adeyi O., Thompson J., Hameed B., Crawford P.A., Ikramuddin S. (2020). Nonalcoholic Steatohepatitis. Gastroenterol. Clin. N. Am..

[149] Tannapfel A., Denk H., Dienes H.-P., Langner C., Schirmacher P., Trauner M., Flott-Rahmel B. (2011). Histopathological diagnosis of non-alcoholic and alcoholic fatty liver disease. Virchows Arch..

[150] Cotter T.G., Rinella M. (2020). Nonalcoholic Fatty Liver Disease 2020: The State of the Disease. Gastroenterology.

[151] Guo X., Yin X., Liu Z., Wang J. (2022). Non-Alcoholic Fatty Liver Disease (NAFLD) Pathogenesis and Natural Products for Prevention and Treatment. Int. J. Mol. Sci..

[152] Harrison S.A., Allen A.M., Dubourg J., Noureddin M., Alkhouri N. (2023). Challenges and opportunities in NASH drug development. Nat. Med..

[153] Hammerich L., Tacke F. (2023). Hepatic inflammatory responses in liver fibrosis. Nat. Rev. Gastroenterol. Hepatol..

[154] Kisseleva T., Brenner D. (2021). Molecular and cellular mechanisms of liver fibrosis and its regression. Nat. Rev. Gastroenterol. Hepatol..

[155] Song Y., Wei J., Li R., Fu R., Han P., Wang H., Zhang G., Li S., Chen S., Liu Z. (2023). Tyrosine kinase receptor B attenuates liver fibrosis by inhibiting TGF-β/SMAD signaling. Hepatology.

[156] Königshofer P., Brusilovskaya K., Schwabl P., Reiberger T. (2019). Animal models of portal hypertension. Biochim. Biophys. Acta Mol. Basis Dis..

[157] Mauro E., Gadano A. (2020). What’s new in portal hypertension?. Liver Int..

[158] Roa J.C., García P., Kapoor V.K., Maithel S.K., Javle M., Koshiol J. (2022). Gallbladder cancer. Nat. Rev. Dis. Primers.

[159] Song X., Hu Y., Li Y., Shao R., Liu F., Liu Y. (2022). Overview of current targeted therapy in gallbladder cancer. Signal Transduct. Target. Ther..

[160] Feo C.F., Ginesu G.C., Fancellu A., Perra T., Ninniri C., Deiana G., Scanu A.M., Porcu A. (2022). Current management of incidental gallbladder cancer: A review. Int. J. Surg..

[161] Brindley P.J., Bachini M., Ilyas S.I., Khan S.A., Loukas A., Sirica A.E., Teh B.T., Wongkham S., Gores G.J. (2021). Cholangiocarcinoma. Nat. Rev. Dis. Primers.

[162] Sarcognato S., Sacchi D., Fassan M., Fabris L., Cadamuro M., Zanus G., Cataldo I., Capelli P., Baciorri F., Cacciatore M. (2021). Cholangiocarcinoma. Pathologica.

[163] Rizvi S., Khan S.A., Hallemeier C.L., Kelley R.K., Gores G.J. (2018). Cholangiocarcinoma—Evolving concepts and therapeutic strategies. Nat. Rev. Clin. Oncol..

[164] Macias R.I.R., Cardinale V., Kendall T.J., Avila M.A., Guido M., Coulouarn C., Braconi C., Frampton A.E., Bridgewater J., Overi D. (2022). Clinical relevance of biomarkers in cholangiocarcinoma: Critical revision and future directions. Gut.

[165] Burgmaier K., Brinker L., Erger F., Beck B.B., Benz M.R., Bergmann C., Boyer O., Collard L., Dafinger C., Fila M. (2021). Refining genotype-phenotype correlations in 304 patients with autosomal recessive polycystic kidney disease and PKHD1 gene variants. Kidney Int..

[166] Goggolidou P., Richards T. (2022). The genetics of Autosomal Recessive Polycystic Kidney Disease (ARPKD). Biochim. Biophys. Acta Mol. Basis Dis..

[167] Kaimori J.-Y., Lin C.-C., Outeda P., Garcia-Gonzalez M.A., Menezes L.F., Hartung E.A., Li A., Wu G., Fujita H., Sato Y. (2017). NEDD4-family E3 ligase dysfunction due to PKHD1/Pkhd1 defects suggests a mechanistic model for ARPKD pathobiology. Sci. Rep..

[168] Klein A.P. (2021). Pancreatic cancer epidemiology: Understanding the role of lifestyle and inherited risk factors. Nat. Rev. Gastroenterol. Hepatol..

[169] Zhang Z., Yu H., Yao W., Zhu N., Miao R., Liu Z., Song X., Xue C., Cai C., Cheng M. (2022). RRP9 promotes gemcitabine resistance in pancreatic cancer via activating AKT signaling pathway. Cell Commun. Signal.

[170] Cai J., Chen H., Lu M., Zhang Y., Lu B., You L., Zhang T., Dai M., Zhao Y. (2021). Advances in the epidemiology of pancreatic cancer: Trends, risk factors, screening, and prognosis. Cancer Lett..

[171] Ben Q., Sun Y., Liu J., Wang W., Zou D., Yuan Y. (2020). Nicotine promotes tumor progression and epithelial-mesenchymal transition by regulating the miR-155-5p/NDFIP1 axis in pancreatic ductal adenocarcinoma. Pancreatology.

[172] Frampton A.E., Castellano L., Colombo T., Giovannetti E., Krell J., Jacob J., Pellegrino L., Roca-Alonso L., Funel N., Gall T.M.H. (2014). MicroRNAs Cooperatively Inhibit a Network of Tumor Suppressor Genes to Promote Pancreatic Tumor Growth and Progression. Gastroenterology.

[173] Su J., Zhou X., Yin X., Wang L., Zhao Z., Hou Y., Zheng N., Xia J., Wang Z. (2017). The effects of curcumin on proliferation, apoptosis, invasion, and NEDD4 expression in pancreatic cancer. Biochem. Pharmacol..

[174] Jiang X., Ma Y., Wang T., Zhou H., Wang K., Shi W., Qin L., Guan J., Li L., Long B. (2023). Targeting UBE2T Potentiates Gemcitabine Efficacy in Pancreatic Cancer by Regulating Pyrimidine Metabolism and Replication Stress. Gastroenterology.

